# Deciphering Epigenetic and Post-Translational Modifications in Ferroptosis: A Scientometric and Visualization Study

**DOI:** 10.7150/ijms.104222

**Published:** 2025-01-01

**Authors:** Siyang Cao, Yihao Wei, Yaohang Yue, Deli Wang, Ao Xiong, Hui Zeng

**Affiliations:** 1 National & Local Joint Engineering Research Centre of Orthopaedic Biomaterials, Peking University Shenzhen Hospital, Shenzhen, Guangdong, People's Republic of China.; 2 Shenzhen Key Laboratory of Orthopaedic Diseases and Biomaterials Research, Peking University Shenzhen Hospital, Shenzhen, Guangdong, People's Republic of China.; 3 Department of Bone & Joint Surgery, Peking University Shenzhen Hospital, Shenzhen, Guangdong, People's Republic of China.; 4 Department of Rehabilitation Science, The Hong Kong Polytechnic University, Hong Kong Special Administrative Region, People's Republic of China.; 5 Faculty of Pharmaceutical Sciences, Shenzhen Institute of Advanced Technology, Chinese Academy of Sciences (CAS), Shenzhen, Guangdong, People's Republic of China.

**Keywords:** epigenetics, post-translational modifications, ferroptosis, scientometrics, visualization analysis

## Abstract

***Background:*** Recent research emphasizes the significant regulatory functions of epigenetic alterations and post-translational modifications (PTMs) in the ferroptosis process. Despite the existing volume of literature, there is a remarkable shortage of comprehensive analyses that systematically trace the evolution of research, map key investigative routes, evaluate the current situation of the field, determine central themes, and predict future directions. This study intends to offer a comprehensive summary of the progress achieved during the past 12 years in comprehending how epigenetic modifications and PTMs regulate ferroptosis.

***Methods:*** The dataset originated from the Web of Science, covering the period from January 1, 2012, to May 21, 2024. By employing advanced analytical tools, we carried out an extensive scientometric assessment in combination with detailed visual data analysis.

***Results:*** The results emphasize the crucial role of China, which contributes 69.59% of the global research output, thereby demonstrating its significant influence on the research trajectory in this domain. Remarkable productivity is manifested at institutions such as Central South University, Shanghai Jiao Tong University, and Zhejiang University. Liu Shuang and Tang Daolin stand out as the most productive authors in this field. The journal *Cell Death & Disease* leads in terms of publication volume, having published the greatest number of articles related to this area. This study identified hepatocellular carcinoma, mitochondrial diseases, and iron overload as the most prominent diseases explored in this research domain.

***Conclusion:*** This meticulous scientometric assessment is beneficial to both experienced researchers and newcomers by providing essential information and facilitating the derivation of innovative concepts in this field.

## Introduction

**Figure 1:**
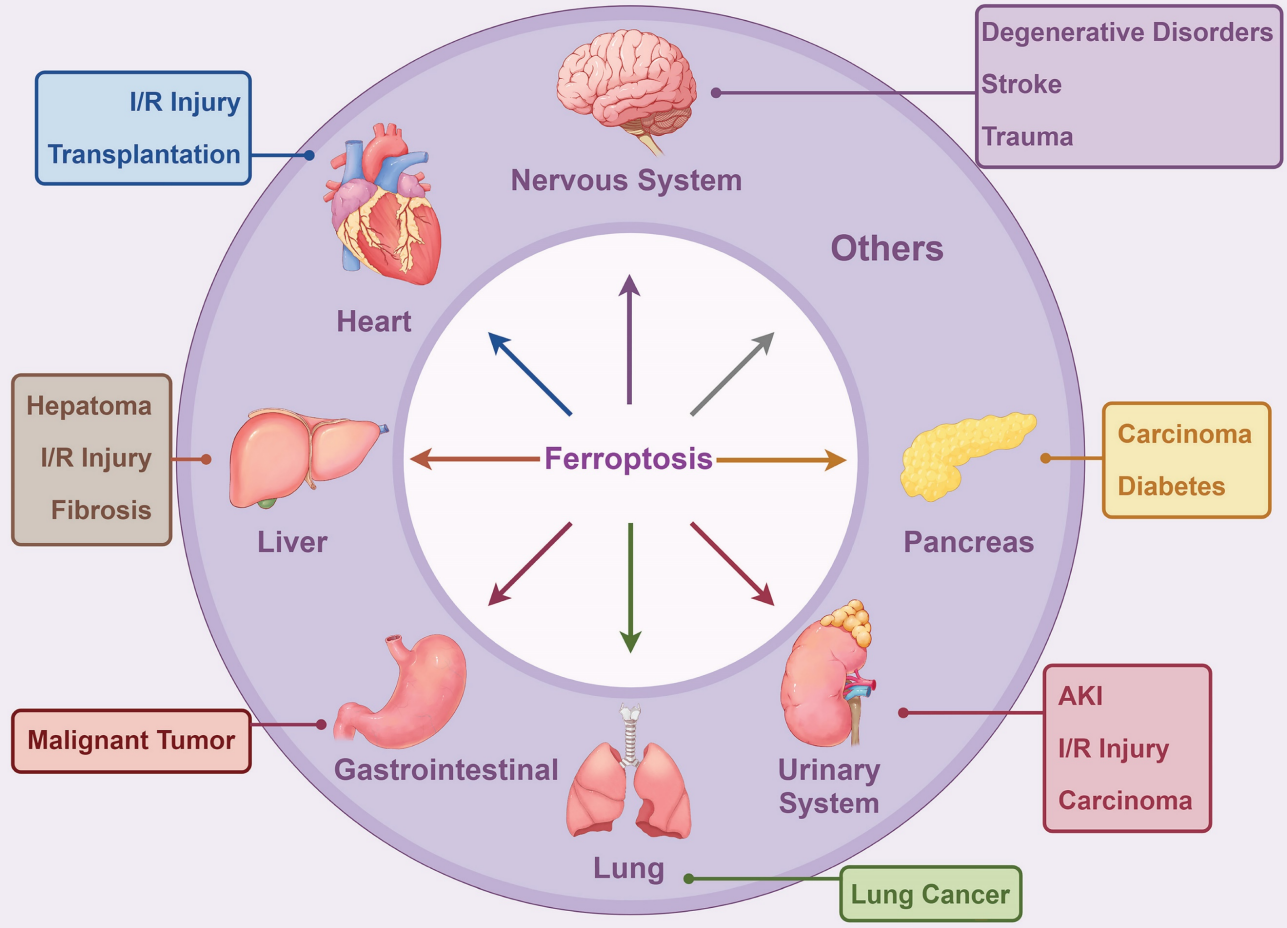
Ferroptosis markedly influences various systemic disorders, including those affecting the nervous, cardiovascular, digestive, respiratory, urinary, endocrine systems, among others. This figure was generated using Figdraw (https://www.figdraw.com/static/index.html#/). **Abbreviations:** AKI, acute kidney injury; I/R Injury, ischemia/reperfusion injury.

Ferroptosis, a novel type of programmed cell death (PCD), is marked by iron-dependent lipid peroxidation (LPO) of polyunsaturated fatty acid-containing phospholipids in cellular membranes^1^-^4^. Its initiation and implementation are regulated by the equilibrium between ferroptosis defense mechanisms^5^. The response to ferroptosis encompasses various metabolic and degradation pathways that regulate LPO or iron accumulation^6^. These pathways include lipid, iron, and amino acid metabolism, along with degradation mechanisms such as the ubiquitin-proteasome system and autophagy. Over the past 12 years, research has increasingly associated ferroptosis with the pathogenesis of a broad range of diseases, affecting nearly every organ system in the body as shown in Figure 1,^7^, ^8^. Exploring the molecular mechanisms and regulatory pathways of ferroptosis might offer novel strategies for treating these conditions.

Emerging research suggests precise regulation of ferroptosis at various levels, involving both protein post-translational modifications (PTMs) and epigenetic alterations^9^, ^10^. These epigenetic mechanisms, including DNA methylation, histone modifications, and regulation through noncoding RNAs, provide dynamic and reversible ways to control gene expression without altering the DNA sequence^11^-^13^. PTMs play a vital role in modifying proteins via enzymatic or covalent alterations, which affect the localization, activity, and interactions at a molecular level^14^, ^15^. These modifications comprise phosphorylation, ubiquitination, SUMOylation, and acetylation^15^. Abnormal epigenetic changes and PTMs can give rise to atypical transcriptional or translational activities, leading to drug resistance, cancer progression, metastasis, and other pathological conditions. These modifications govern ferroptosis-related gene expression, influencing cancer cell susceptibility to ferroptosis at both transcriptional and translational stages^9^, ^16^-^18^. Recent studies have emphasized the crucial role of epigenetic alterations and PTMs in regulating ferroptosis in various medical conditions, including neurological disorders, cardiovascular diseases, hepatic conditions, pulmonary disorders, and renal diseases^19^.

Science mapping studies often utilize scientometric tools, metrics, and indicators to explore scientific literature, identifying and analyzing pathways, trends, and theories related to scientific evolution. We conducted a comprehensive exploration of how epigenetic modifications and PTMs influence ferroptosis, employing both scientometric and statistical methods. In contrast to previous traditional reviews^10^, ^16^, ^19^-^27^, this article presents empirical evidence supported by objective and visual data. This considerably reduces the possibility of subjective bias among researchers and lowers the degree of variability. This approach facilitates a more thorough analysis and helps delineate the current research landscape. Furthermore, the study contributes to the existing literature by providing an in-depth analysis and visualization of data through scientific mapping tools such as CiteSpace and VOSviewer. The scientometric analysis indicates both the most and least investigated areas in the discipline, identifying topics that require further research and suggesting potential collaboration opportunities for future research projects. This analysis promotes better communication among groups focusing on similar fundamental issues.

This study aims to explore a set of crucial research questions (RQs) designed to enhance our understanding of the field: RQ1: What are the dominant trends in recent research on "epigenetic modifications and PTMs in ferroptosis"? RQ2: Which countries, institutions, and researchers have the most substantial influence in this area of study? RQ3: What publications, references, and keywords are most frequently cited and essential to this discipline? RQ4: Which genes and signaling pathways are centrally implicated? RQ5: What related diseases are most prominent? The study employs a mixed-methods approach, integrating quantitative and qualitative analyses. The quantitative part encompasses an examination of research themes, publication years, languages, journal scopes, and metadata such as authorship, geographic distribution, institutional affiliations, journal venues, and citation metrics. The qualitative aspect focuses on the thematic mapping of keywords. The research examines the current progress status, primary contributors, top countries and journals, publication trends, existing comprehension, and potential research directions in epigenetic changes and PTMs within ferroptosis. The establishment of a systematic and comprehensive knowledge repository is beneficial for researchers from diverse disciplines to grasp the scope of this domain and offers a structured roadmap for newcomers, guiding them toward productive research trajectories. According to our review, there are no current scientometric studies that have specifically concentrated on this topic.

## Materials & Methods

### Data Source & Retrieval Strategy

The Web of Science Core Collection (WoSCC) (https://www.webofscience.com/wos/) is a vital resource for tracking scientific progress and conducting in-depth analyses of scholarly publication trends^28^-^31^. The WoSCC provides strong scientometric tools that are indispensable for generating general statistical insights^30^ and offers better document type categorization than other databases^32^. In this research, we used the WoSCC to conduct a comprehensive online search for original research articles and review papers related to "epigenetic modifications and PTMs in ferroptosis". This search covered publications from January 1, 2012, to May 21, 2024, using both Medical Subject Headings (MeSH) terms and free words. The search methodology was repeatedly refined by a team of three researchers to improve both sensitivity and precision, with a detailed description provided in the Supplementary Materials.

### Inclusion & Exclusion Standards

Special attention was paid to studies and reviews published in English that concentrate on "epigenetic modifications and PTMs in ferroptosis". Dissertations, letters, comments, editorials, and conference abstracts were not taken into consideration in the analysis. Also excluded were duplicate studies which were published with the same titles in various journals. Team members and peer groups had in-depth discussions about the inclusion and exclusion criteria.

### Data Visualization & Analysis

Data from the WoSCC database was collected and imported into Microsoft Excel (Office 365, Microsoft, USA). Analysis was conducted using VOSviewer 1.6.18 (Leiden University, Netherlands), Citespace 6.3.R1 (Chaomei Chen, China), Pajek 5.16 (University of Ljubljana, Slovenia), Scimago Graphica 1.0.35 (https://www.graphica.app/, USA), and the chorddiag R package (R Studio, version 4.2.0).

The chorddiag R package and VOSviewer were employed to generate visual depictions of national and regional research collaborations and to carry out analyses of the published literature corpus. Co-occurrence analyses, covering countries/regions, institutions, authors, journals, and diseases, were conducted using VOSviewer, Scimago Graphica, and Pajek. Citespace enabled the visual representation and analysis of data related to countries/regions, institutions, authors, journals, co-citations, and keywords. Temporal trends in keyword popularity were investigated using Scimago Graphica. Additionally, the chorddiag R package was used to portray the overall publication landscape. Gene Ontology (GO) and Kyoto Encyclopedia of Genes and Genomes (KEGG) pathway enrichment analyses were conducted using the ggplot2, enrichplot, and clusterprofiler R packages, offering a detailed inspection of biological pathways and processes.

Disease-related information was obtained from the Citexs Data Analysis Platform, accessible at https://www.citexs.com. This platform simplifies the process of creating visualizations, thereby facilitating a comprehensive examination of the current condition, key research areas, and emerging trends within this scientific field.

## Results & Discussion

### Annual Publications

**Figure 2:**
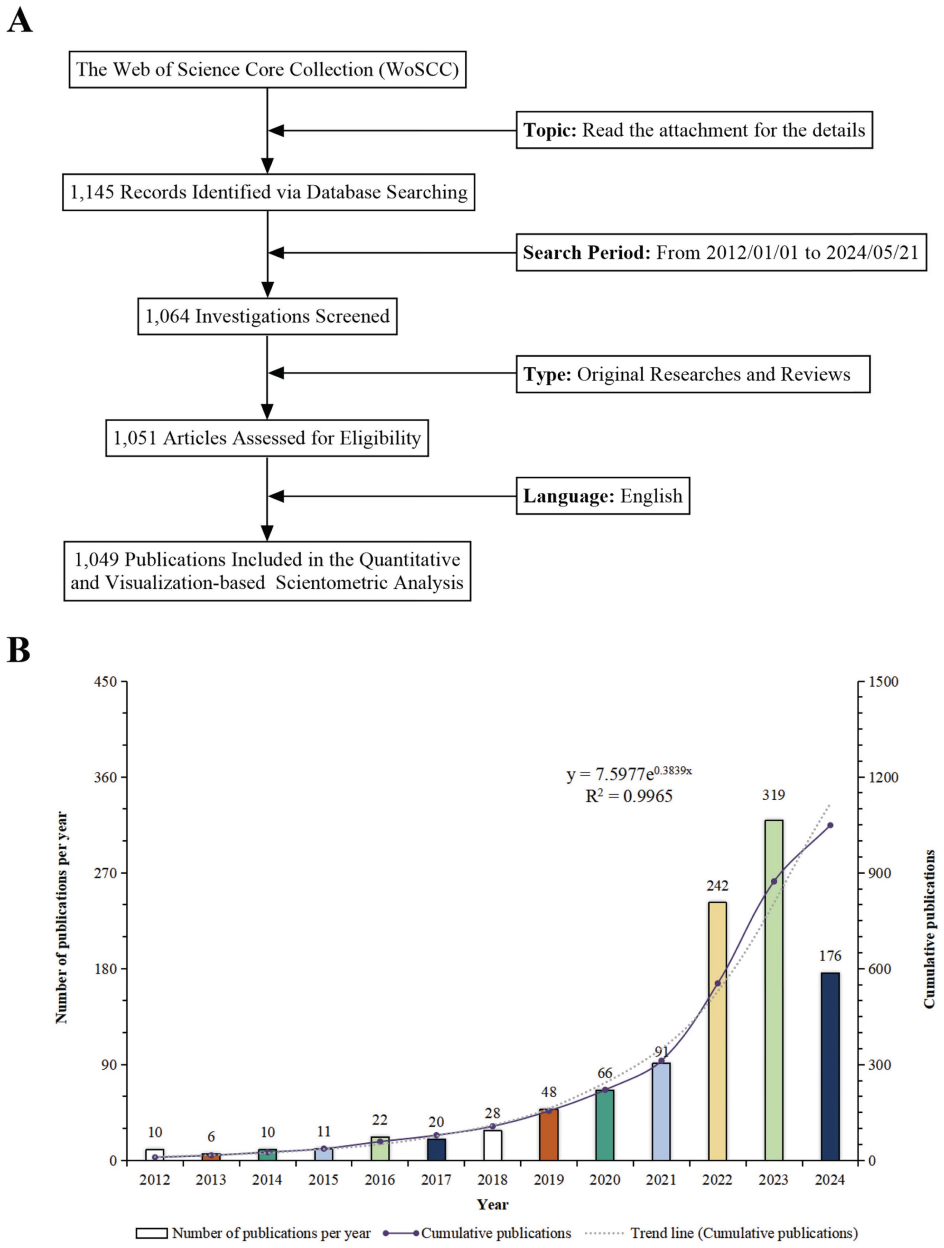
(A) Schematic presentation of the methodology for literature search and selection. (B) Temporal trend analysis demonstrating the development of research on epigenetic and post-translational modifications in ferroptosis from 2012 to 2024.

Figure 2A depicts the process of data retrieval and collection. The trajectory of research advancement is measured by monitoring the quantity of scientific publications over a specified period^33^. From 2012 to 2024, a total of 1,049 scientific publications related to "epigenetic modifications and PTMs in ferroptosis" were documented, consisting of 879 original articles and 170 reviews. This amounts to an average annual publication rate of 87.42, indicating strong interest in this domain. In 2022, the number of annual publications exceeded 200, reaching a peak of 319 publications in 2023. The diagram shows a 31-fold increase since 2012, mirroring a growing research community and improved prospects for collaboration, innovation, and funding. The exponential growth trend (y = 7.5977e^0.3839x^), where x represents the year and y the number of publications (Figure 2B), describes the annual publication trajectory. The high coefficient of determination (R² = 0.9965) suggests a robust analysis, offering a solid foundation for future research predictions and strategic planning. The graph forecasts a continuous rise in annual research output, emphasizing the growing interest in the study of "epigenetic modifications and PTMs in ferroptosis". Consequently, considerable progress is expected in this field over the next few years.

The escalating research on "epigenetic modifications and PTMs in ferroptosis" indicates its significance and potential for substantial scientific and therapeutic progress. This advancement benefits researchers, industry practitioners, policymakers, and educators, emphasizing its multidisciplinary influence. Industry practitioners, particularly in biotechnology and pharmaceuticals, can utilize insights regarding epigenetic modifications and PTMs to develop novel therapeutics targeting ferroptosis-related pathways. The vigorous growth implies fertile terrain for public-private partnerships and commercialization opportunities. Policymakers can advocate policies that support continuous research funding and infrastructure development to promote innovation. Substantial scientific progress can assist in prioritizing research agendas and informing health policies. The rise in publications implies an abundance of new knowledge and resources for educators and students. This expanding corpus of work can enrich curricula, offer case studies, and stimulate new research projects. Students entering this field have copious opportunities for discovery and contribution to a rapidly evolving area of science.

### Countries/Regions

**Figure 3:**
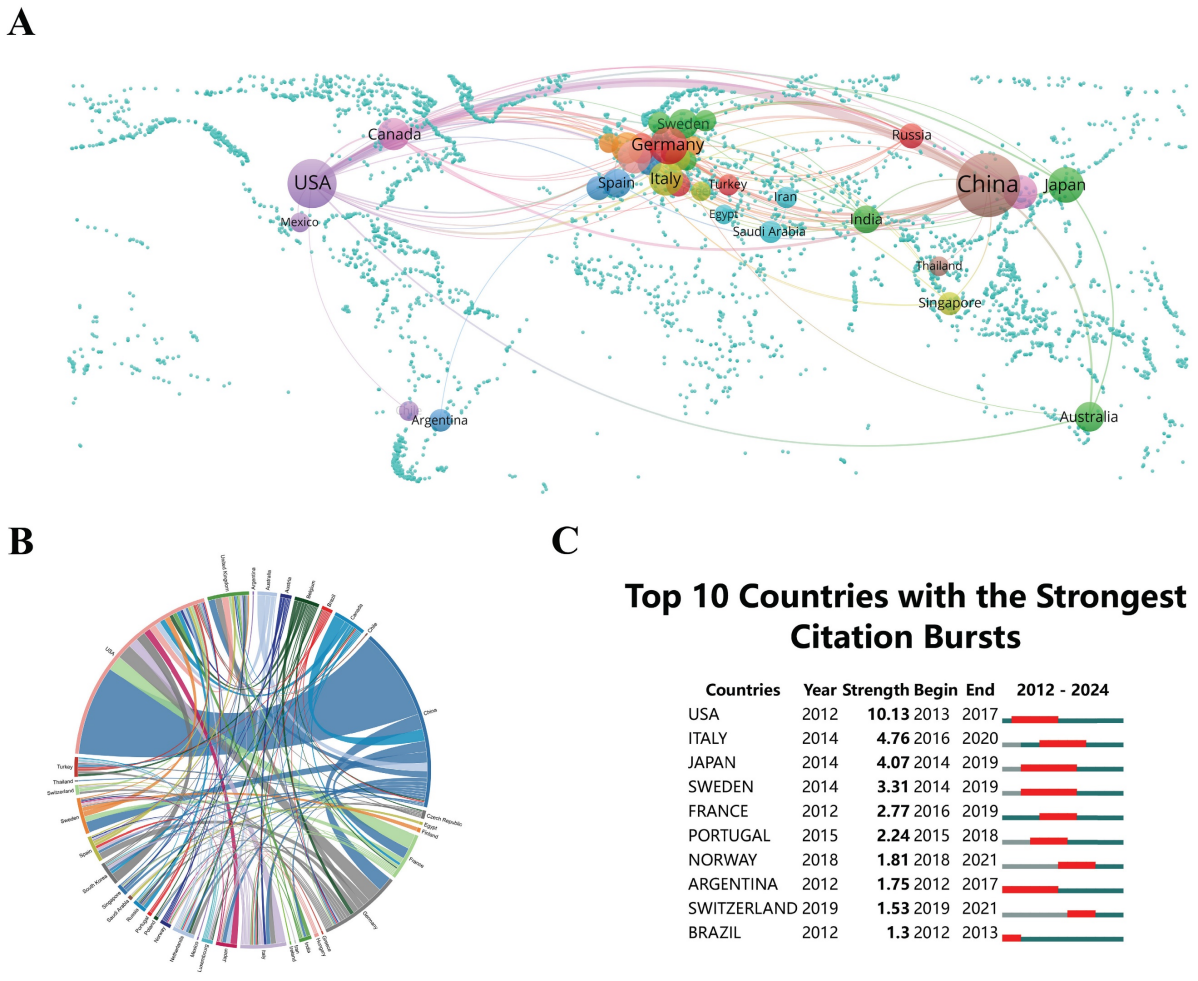
Global distribution of research on epigenetic and post-translational modifications in ferroptosis. Each sphere represents a country, with the thickness of the lines signifying the levels of collaboration. The sizes of the spheres correlate to the number of publications. (B) Chord diagrams of international collaborations, where each outer curve represents a country, and the thickness of the lines indicates the strength of collaboration. (C) Research output of the top 10 countries, with increased document production highlighted in red.

Global research on "epigenetic modifications and PTMs in ferroptosis" covers 51 countries/regions, establishing a comprehensive and collaborative environment for researchers. The national collaboration networks in Figure 3A and 3B promote strategic partnerships, enhancing research quality and innovation, with each country contributing at least two publications. This offers essential perspectives for strategic collaborations and knowledge exchange. Notably, China's 730 publications emphasize its prominent position in the field and indicate extensive opportunities for collaborative research. Researchers in the United States (18.30%, 192 publications) and Germany (4.96%, 52 publications) also make significant contributions, facilitating knowledge exchange and progress in the field. In the chord diagram, the peripheral curve segments visually demarcate each country and region. The size of each segment is proportionally associated with the publication output of the respective country or region. Furthermore, the level of connectivity between nations within the diagram demonstrates the extent of their collaborative interactions. The United States has the highest frequency of global collaboration, mainly cooperating with China (link strength = 66) (Figure 3B). This knowledge can guide strategic decisions regarding investments, partnerships, and innovations^34^.

The identification of publications undergoing significant citation increments over time is crucial and is facilitated through the analysis of citation bursts. Figure 3C depicts the citation spikes for the top 10 countries/regions, where the intensity of the red line is directly correlated with the magnitude of these bursts. From 2013 to 2017, the United States witnessed a considerable citation growth, attaining the highest burst strength value of 10.13. This helps researchers in identifying influential studies and emerging trends, guiding future research. Japan, Sweden, and Argentina exhibited the longest citation burst durations, each lasting for five years, indicating their continuous academic influence. The majority of the top 10 countries underwent citation bursts before 2019, suggesting that global interest and research activity in this field began escalating before 2019.

Global research on "epigenetic modifications and PTMs in ferroptosis", led by China and with significant contributions from the United States and Germany, offers valuable perspectives for researchers, industry practitioners, policymakers, educators, and students. The increasing collaboration and citation bursts signify a vigorous and expanding research community, providing numerous opportunities for partnerships, innovation, and advancements in this field. The leadership of China, along with significant contributions from the United States and Germany, emphasizes the need for policies that encourage cross-border collaborations and knowledge sharing. Policymakers can advocate sustained research funding and infrastructure development to boost their countries' academic influence. The growth in research publications and international collaborations supplies educators with ample resources for curriculum development and case studies. Students entering this field have access to a dynamic and evolving body of knowledge, presenting numerous research opportunities and innovations. Identifying countries with significant citation bursts assists educators in highlighting impactful studies and emerging trends, inspiring students and guiding their academic pursuits. The vigorous growth and global cooperation in this research area ensure a promising future for aspiring scientists.

### Institutions & Partnerships

**Figure 4:**
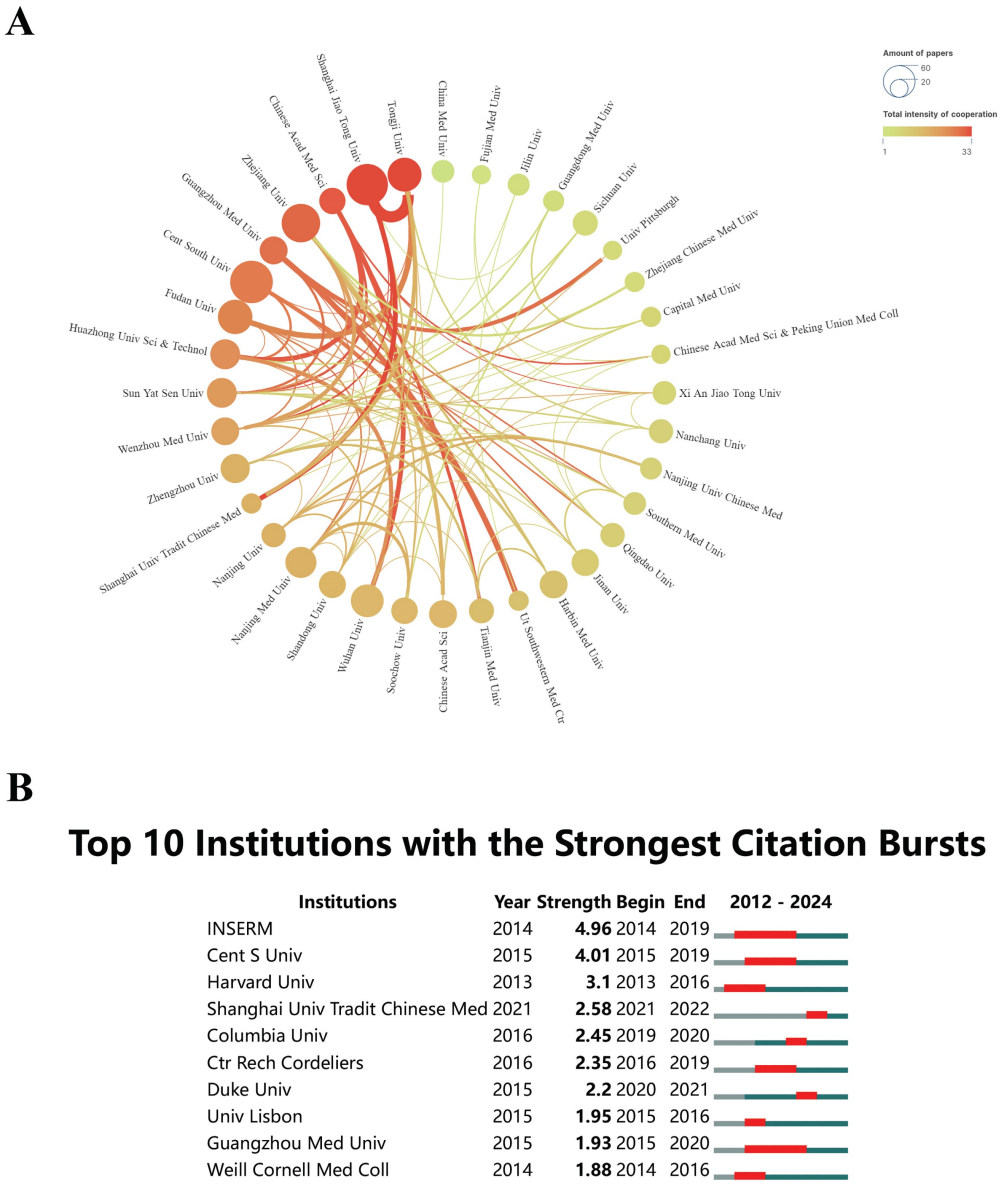
(A) Diagram depicting the intensity of institutional cooperation, where the thickness of the lines between the circles indicates the cooperation levels. The size of the circles is in proportion to the number of documents issued by each institution. (B) Citation bursts of the top 10 institutions, represented by red bars, signify periods of increased citation activity.

The analysis of leading organizations and their citation bursts provides crucial insights for researchers seeking potential collaborators. The dynamism within these collaborative networks indicates a strong research ecosystem, which is helpful in enhancing research quality and fostering innovation. Over the past 12 years, global research on "epigenetic modifications and PTMs in ferroptosis" has made significant progress, involving more than 1,330 entities. Figure 4A presents a network of collaboration among different institutions, which demanded a minimum of 10 publications for participation. The network comprises circles and text representing individual institutions, with connecting lines denoting collaborative instances. In the visualization, the thickness of the lines indicates the degree of collaborative interactions, where thicker lines indicate stronger partnerships. The gradient colors used to shade the lines signify the overall level of collaboration between institutions, where a more intense color implies a higher degree of interaction. Additionally, the size of the circles is related to the publication volume, with larger circles representing institutions that have generated a higher number of publications. Central South University stands out as the leading contributor, accounting for approximately 4.67% (49 publications) of the overall research output in this field. Close behind are Shanghai Jiao Tong University (4.39%, 46 publications) and Zhejiang University (3.81%, 40 publications). Institutions have made remarkable advancements in this field, making them potential candidates for future research collaboration. Our assessment indicates that Shanghai Jiao Tong University and Tongji University have shown a considerable tendency to collaborate with other institutions. Importantly, most of these organizations prioritize domestic collaborations over international partnerships.

Identifying institutions experiencing citation surges helps researchers in identifying active and influential research programs. These programs might provide opportunities for collaboration or funding. When evaluating potential collaborations, it is crucial to consider not only the publication count but also the sustained influence and adaptability of the research over time^35^, ^36^. By using CiteSpace analysis (Figure 4B), this study identified institutions with remarkable citation bursts. Institut National de la Santé et de la Recherche Médicale (INSERM) underwent prolonged citation bursts from 2014 to 2019. Nevertheless, this trend has declined over the past five years. The citation burst duration for Guangzhou Medical University, from 2015 to 2020, is the same as that of INSERM, both lasting for five years. The prolonged citation bursts of institutions such as INSERM and Guangzhou Medical University suggest sustained academic influence in the field of "epigenetic modifications and PTMs in ferroptosis". Researchers can collaborate with these institutions to increase the impact and reach of their work. In the past three years, Shanghai University of Traditional Chinese Medicine has demonstrated a significant increase in citations, suggesting that its publications have recently received substantial attention in this research field. These findings emphasize the significance of strategic collaborations, funding opportunities, and understanding global research trends in advancing the field of "epigenetic modifications and PTMs in ferroptosis". Industry practitioners and researchers can make use of this data to enhance their potential for innovation and impact in research.

### Core Authors

**Figure 5:**
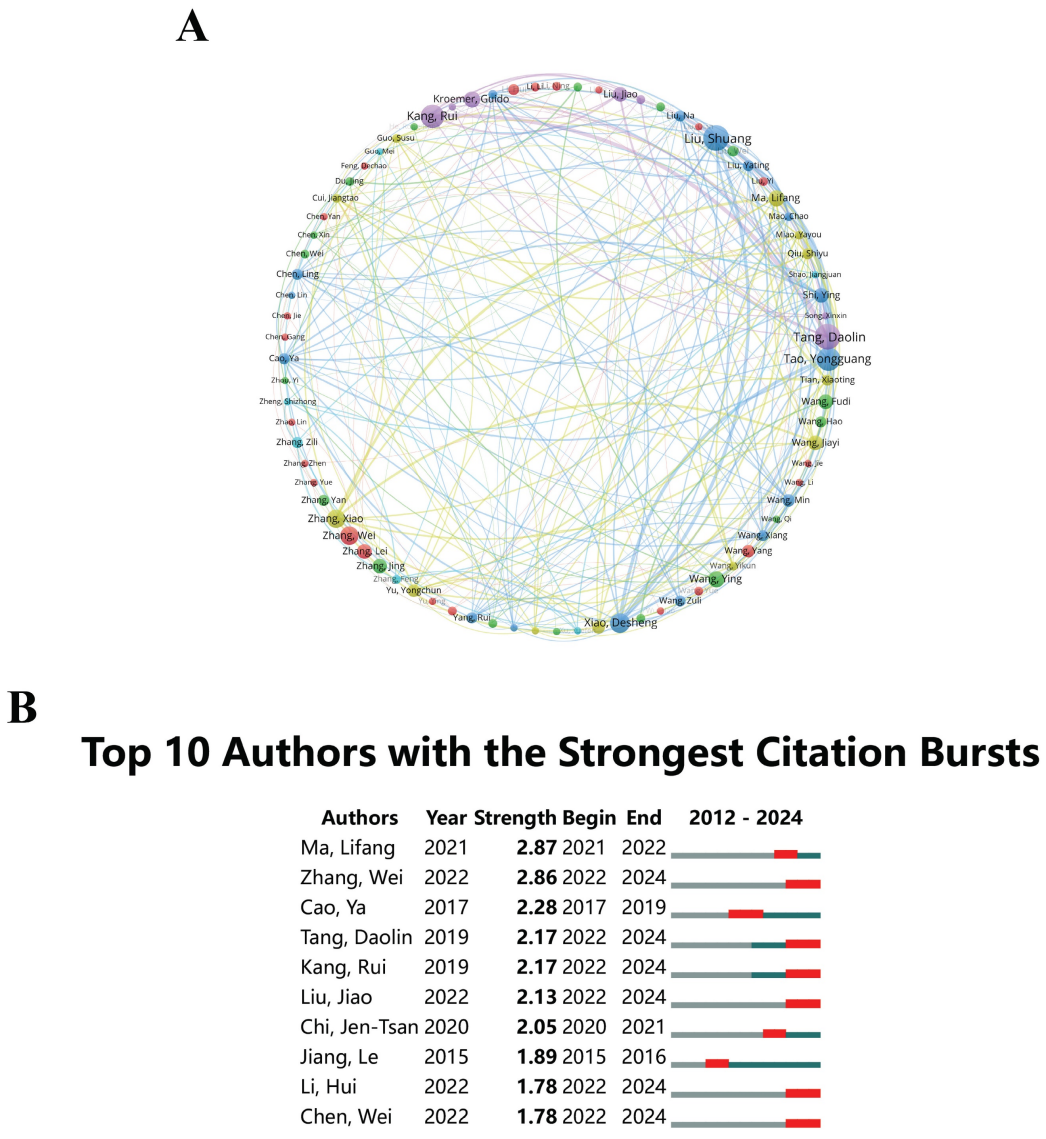
(A) Co-occurrence map of authors, where nodes are constituted by circles and text labels, and distinct clusters are signified by different colors. (B) The top 10 authors with the most significant citation bursts in publications related to epigenetic and post-translational modifications in ferroptosis.

Identifying prominent researchers and exploring their collaborative networks enables scholars to identify the key contributors within the discipline. Researchers who display significant citation counts and maintain a consistent publication record offer critical insights and assist in shaping the direction of future research. A comprehensive analysis of authorship in the domain of "epigenetic modifications and PTMs in ferroptosis" determined 7,198 significant contributors. Notably, seven researchers were particularly prominent, each having authored at least 10 articles. These individuals probably possess extensive expertise, making their contributions extremely valuable for those exploring this research area. For a comprehensive analysis of co-authorship networks, the VOSviewer software was utilized to create visual representations. These visualizations were generated with a set threshold demanding a minimum of four publications per researcher. In these visual depictions, the diameter of each circle is directly correlated with the publication count attributed to each author. Different colors represent different clusters of authors, and the thickness of the lines connecting the circles demonstrates the degree of collaboration between them. Notably, 80 authors met this threshold. Tang Daolin and Kang Rui exhibited the strongest collaborations, as depicted in Figure 5A. Additionally, Liu Shuang and Tang Daolin, each with 14 publications, were recognized as highly productive scholars in this field, highlighting their essential contributions. Insights into prolific authors and their collaborations offer valuable guidance for emerging researchers and students seeking mentorship and leaders in this field.

Understanding citation dynamics and identifying influential authors provide a clear perspective of impactful research, helping researchers prioritize their reading and integrate significant findings. Citation burst analysis measures the rate at which an author's work is cited within a designated research area over a specified period^37^, ^38^. Figure 5B depicts the top ten researchers with the highest citation counts in the field of “epigenetic modifications and PTMs in ferroptosis”. Significantly, Ma Lifang shows a citation burst strength of 2.87, which differentiates her from her peers, with Zhang Wei and Cao Ya also presenting notable citation impacts. This analysis is helpful in identifying emerging trends and shifts in research emphasis, ensuring that researchers and practitioners remain informed about the latest developments. For industry practitioners and funding bodies, detailed authorship and collaboration analysis assists in identifying key researchers and groups to support, ensuring that investments are targeted at leading experts and promising research clusters.

It must be noted that the number of papers published by scholars is not a fair and objective metric for assessing their contribution to a specific field. It is merely a quantitative measurement. Some scholars might excel in in-depth research, and their research outcomes are highly valuable. Nevertheless, due to the nature of the research and other factors, the number of papers they generate is restricted. Furthermore, in the current academic milieu, there are some low-quality studies carried out for the sake of the number of papers published. Some scholars might split a research result into multiple papers for publication or undertake repetitive research when there is a lack of innovation. This is particularly conspicuous in the context of predatory journals, where a considerable number of published papers do not necessarily indicate a significant contribution to the field^39^, ^40^. We should approach this issue dialectically, which implies that we should not only take into account some reference value that the number of papers published might bring, such as reflecting the scientist's activity and the continuity of research to a certain degree, but also not regard it as the sole measurement criterion. We need to be aware that scientific contribution is a multi-dimensional notion, encompassing theoretical innovation, technological application, leadership in the development direction of the discipline, talent nurturing, and many other aspects. With the escalating complexity and diversity of scientific research, the existing single-index evaluation is hard to fulfill the requirements of accurately assessing the contribution of scholars. A flawless evaluation system might need to comprehensively contemplate multiple factors such as the influence of research results (like the number of citations and the value in practical applications), the innovation of the research (whether it has initiated a new research direction, whether it has resolved long-standing issues), and the role played by scholars in academic exchanges and cooperation. Altering the evaluation method that overly depends on the number of papers published is conducive to creating an outstanding scientific research ecology. If solely guided by the number of papers published, it might lead researchers to be eager for instant success and disregard the quality and depth of the research. The adoption of a comprehensive evaluation system can stimulate scholars to pay greater attention to the substantive contribution of the research and undertake more meaningful explorations^41^.

### Main Sources Journals

**Figure 6:**
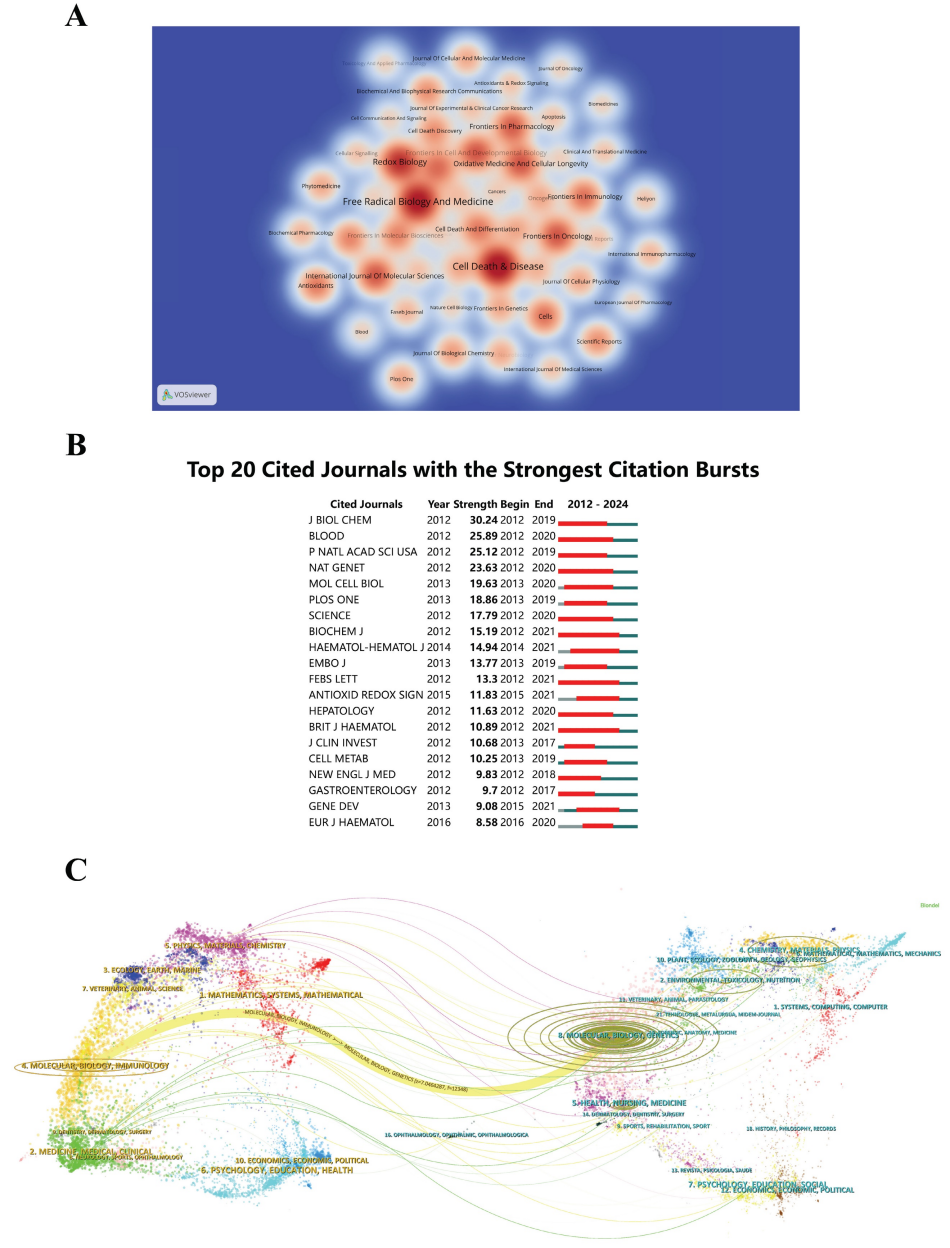
(A) Density visualization map of journal citations, where the color intensity is correlated with the publication volume. (B) The top 20 journals with the most significant citation bursts. (C) Dual-map overlay of journals related to epigenetic and post-translational modifications in ferroptosis. Each point represents a journal, and curves denote citation connections. These paths illustrate interdisciplinary relationships and citation trends.

Analyzing the visualized journal publication data shows that 425 journals have published articles on this topic. To describe the distribution of documents across various journals, a heatmap was created (Figure 6A), using a threshold minimum of six documents per journal. '*Cell Death & Disease*' ranks the highest with 30 documents (2.86%), followed by '*Free Radical Biology and Medicine*' with 28 (2.67%), and '*Redox Biology*' with 22 (2.10%). This assists researchers in targeting high-impact journals, enhancing the visibility and dissemination of their work. Additionally, Figure 6B shows the top 20 journals presenting the most significant citation spikes for these works. Identifying journals with the strongest citation bursts and classifying research into key domains offers insights into emerging trends and shifts in focus. This enables researchers and practitioners to stay updated with the latest developments and adjust their research priorities.

The dual-map overlay technique shows the distribution of journals from different disciplines, the progress of citation paths, and the changing positions of research hubs^42^. The map labels indicate the specific subject areas of these journals. On the left are journals that cite others, while on the right are those being cited^43^. The colored lines on the map denote citation pathways, with the thickness of these lines corresponding to the frequency of *z*-score-normalized citations. Figure 6C categorizes research on "epigenetic modifications and PTMs in ferroptosis" into two main domains: molecular biology and immunology. These insights from diverse disciplines might promote cooperation among researchers across various fields, facilitating the sharing of ideas and stimulating creativity. For industry practitioners, comprehending the publication landscape and citation dynamics assists in identifying leading journals and influential research trends. This steers investment in cutting-edge research and collaboration with key academic partners. Funding bodies can utilize detailed publication and citation data to allocate resources effectively, supporting influential research in "epigenetic modifications and PTMs in ferroptosis". This guarantees optimal funding use for significant progress.

### Co-cited References

**Figure 7:**
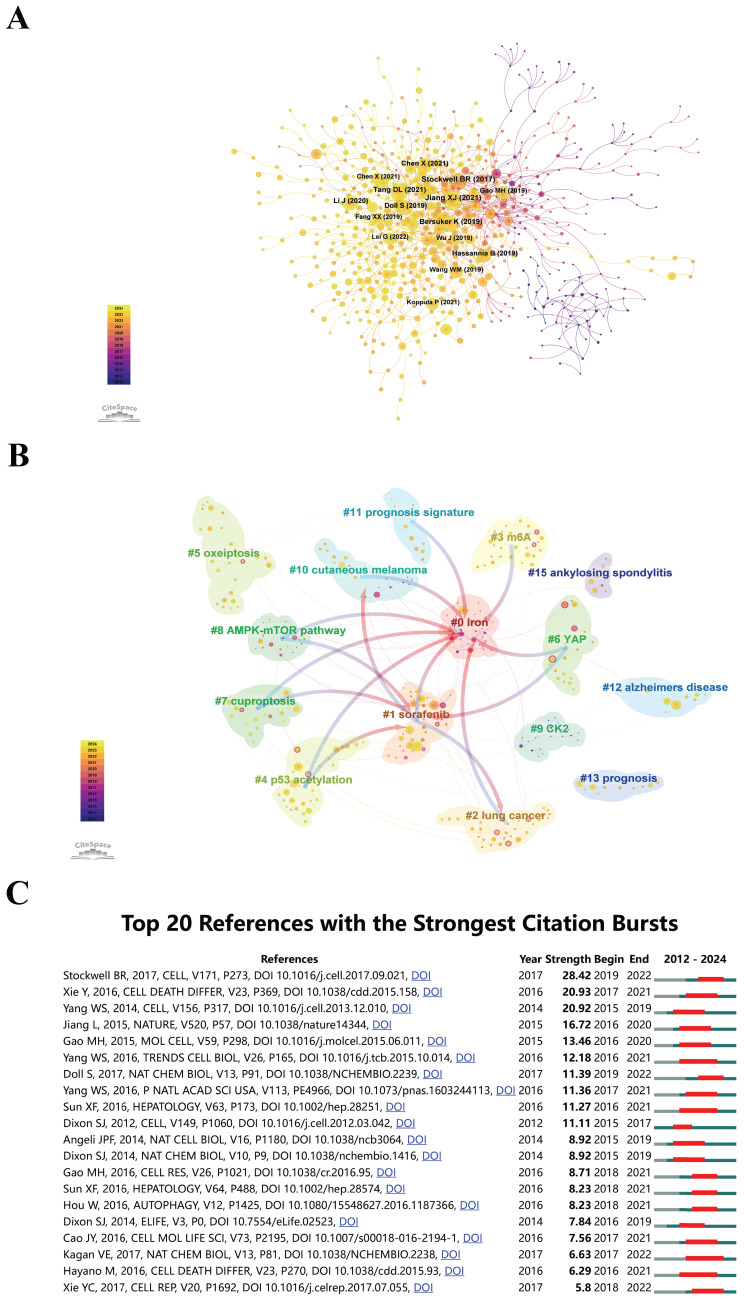
(A) Co-citation analysis chart of epigenetic and post-translational modifications in ferroptosis. The sizes of the circles, similar to annual rings, represent citation counts. Purple denotes earlier citations, yellow denotes later ones, and overlapping colors signify citations in the same years. Lines depict co-citation patterns, with magenta nodes highlighting critical nodes with centrality exceeding 0.1. (B) Co-cited literature network map. The superimposed sphere sizes correspond to co-citations. Purple indicates earlier citation times, yellow indicates later citation times, and overlapping colors signify citations in multiple years. Lines represent co-citation relationships. Magenta nodes are key nodes with centrality exceeding 0.1. (C) The top 20 references with the most salient citation burst.

It is crucial for researchers to identify the most cited works to enhance their comprehension and further promote their fields. Figure 7A presents a co-citation network analysis of publications related to "epigenetic modifications and PTMs in ferroptosis", covering the period from January 1, 2012, to May 21, 2024. This analysis was carried out using the CiteSpace software. This contributes to developing comprehensive literature reviews and identifying key research trends. In this visualization, the size of the spheres, accumulated across the annual rings, reflects their co-citation frequencies. The color gradient shifts from purple, representing earlier citations, to yellow, indicating more recent citations. Spheres showing overlapping colors signify sustained citation activity over time. The connecting lines between spheres demonstrate co-citation relationships among various publications. Nodes highlighted in magenta indicate key points within the network, distinguished by a centrality score greater than 0.1. The article titled "Ferroptosis: Mechanisms, Biology, and Role in Disease" by Jiang Xuejun et al., published in *Nature Reviews Molecular Cell Biology* in 2021, is ranked as the most frequently co-cited publication, with a co-citation count of 124^44^. Closely following is the 2020 article "Ferroptosis: A Regulated Cell Death Nexus Linking Metabolism, Redox Biology, and Disease" by Brent R. Stockwell et al., published in *Cell*, which has obtained 116 co-citations^1^.

CiteSpace employs metrics such as Modularity (*Q* score) and Mean Silhouette (*S* score) to evaluate the structure of networks and the clarity of clustering. A *Q* score exceeding 0.3 indicates the presence of significant clustering, while an *S* score above 0.5 implies that the clusters are well-defined and effectively formed. In this analysis, the calculated scores were *Q* = 0.8561 and *S* = 0.9582, which highlight the robustness of the network and increase confidence in cross-disciplinary research collaborations. The analysis uncovered 15 distinct clusters, designated as #0 iron, #1 sorafenib, #2 lung cancer, #3 m6A, #4 p53 acetylation, #5 oxeiptosis, #6 YAP, #7 cuproptosis, #8 AMPK-mTOR pathway, #9 CK2, #10 cutaneous melanoma, #11 prognosis signature, #12 Alzheimer's disease, #13 prognosis, and #15 ankylosing spondylitis, as shown in Figure 7B. Identifying these 15 distinct clusters provides a roadmap for emerging research areas, ensuring that researchers can align their work with significant and well-cited topics.

Citation burst analysis assists researchers in prioritizing reading materials and integrating significant findings into their work, enhancing its quality and relevance. CiteSpace's analytical tools were utilized to detect citation spikes, focusing on research that has received considerable interest in "epigenetic modifications and PTMs in ferroptosis". In Figure 7C, an examination of the top 20 citations reveals their considerable influence through notable citation bursts. The increase in citations since 2015 indicates growing interest and advancements in this field, which can assist in shaping future research priorities and strategic planning. In 2016, 30% (6 out of 20) of the references exhibited citation bursts, making it the year with the highest frequency of these occurrences. This was followed by the years 2015, 2017, and 2018, each accounting for 20% (4 out of 20) of the bursts. The research paper that exhibited the most remarkable citation burst (strength = 28.42) was 'Ferroptosis: A Regulated Cell Death Nexus Linking Metabolism, Redox Biology, and Disease', which was published in *Cell*^1^. This was closely succeeded by the studies conducted by Xie et al^45^, and Wan Seok Yang et al.^46^. Funding bodies can utilize detailed co-citation and citation burst analyses to identify high-impact research areas and allocate resources to support the most influential and emerging topics. This guarantees that funding is directed towards research with the highest potential for scientific advancement and practical application.

### Relevant Keywords

**Figure 8:**
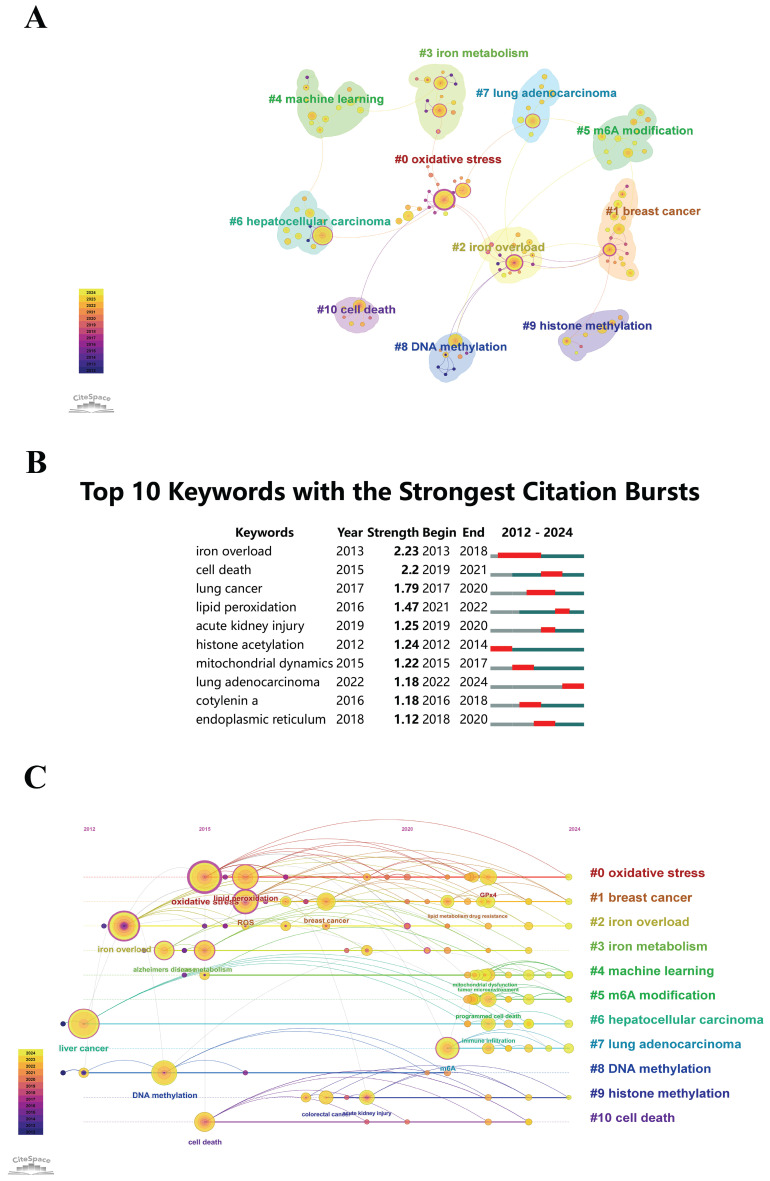
(A) Clustering analysis of keyword frequencies. The sizes of the circles, corresponding to the sizes of annual rings, are proportional to co-citations. Purple indicates earlier citation times, yellow indicates later ones, and overlapping colors signify citations in corresponding years. Lines represent keyword co-citation, with magenta nodes marking critical nodes with centrality exceeding 0.1. (B) The top 10 keywords with the most powerful citation bursts identified by CiteSpace. (C) Temporal trends in keyword co-occurrence. The sizes of the circles on the annual rings are proportional to the keyword frequency. Lines represent co-occurrence. Purple indicates the early appearances of keywords, yellow indicates later ones, and overlapping colors denote the appearances in corresponding years. Magenta nodes, positioned centrally, indicate strong centrality nodes acting as hubs. Keywords within the same cluster are horizontally aligned. The top indicates the first appearance time of keywords, progressing towards the right.

CiteSpace evaluates the quality of network structures and clustering clarity through metrics such as Modularity (*Q* score) and Mean Silhouette (*S* score). A *Q* score exceeding 0.3 implies significant clustering, while an* S* score above 0.5 indicates well-defined and efficient clusters. In this analysis, the network yielded a Modularity (*Q*) score of 0.8413 and a Mean Silhouette (*S*) score of 0.9483, affirming the presence of robust and coherent clustering patterns. The inspection identified 11 distinct clusters: #0 oxidative stress, #1 breast cancer, #2 iron overload, #3 iron metabolism, #4 machine learning, #5 m6A modification, #6 hepatocellular carcinoma, #7 lung adenocarcinoma, #8 DNA methylation, #9 histone methylation, and #10 cell death, as shown in Figure 8A. This clustering enables researchers to identify emerging trends and gaps in the literature, guiding future research directions. It also assists in understanding the interconnectedness of different research areas, leading to more comprehensive and multidisciplinary studies.

Figure 8B depicts the detection of keyword bursts, especially those showing substantial citation surges, thereby highlighting topics that have received significant academic attention. Analyzing the temporal dynamics of these keyword bursts offers valuable perspectives for researchers, funding agencies, and industry stakeholders, informing potential investment and collaboration opportunities. Such analyses also ease alignment with current research trends and the formation of strategic partnerships. Notably, Figure 8B indicates that the keyword 'iron overload' had the highest citation burst intensity, with a value of 2.24, and the longest duration, spanning from 2013 to 2018. Among the top 10 keywords, "iron overload" and "histone acetylation" underwent early citation bursts, indicating early attention to these research hotspots. Keywords emerging in citation bursts after 2022, as identified in the analysis, include "lung adenocarcinoma", indicating its emergence as a key focus area in recent years. By concentrating on the most cited and emerging topics, industry practitioners can prioritize their investments and collaborations, ensuring they work on cutting-edge areas with high potential for innovation and impact. This alignment can speed up the translation of research findings into practical applications and new technologies.

Analyzing the evolving trends and key research areas within "epigenetic modifications and PTMs in ferroptosis" is crucial for guiding funding decisions, promoting collaborations, and aligning with current research priorities. Figure 8C presents a chronological timeline that depicts the clustering of keyword frequencies across significant research domains. The diagram has circles of different sizes, where each circle's diameter is in proportion to the frequency of keyword occurrences in each corresponding year. Connections among keywords indicate their co-occurrence. Nodes highlighted in rose color denote critical keywords of central importance, emphasizing their crucial roles as focus points within the network. The arrangement of keywords within each cluster is displayed horizontally, with the earliest occurrences on the left and subsequent keywords moving towards the right, indicating the passage of time. This visualization eases the understanding of keyword distribution across clusters, with the size of the nodes representing the relative significance of each keyword. It also makes clear the temporal progression of keyword prominence. The identified keywords are categorized into 11 distinct clusters: #0 oxidative stress, #1 breast cancer, #2 iron overload, #3 iron metabolism, #4 machine learning, #5 m6A modification, #6 hepatocellular carcinoma, #7 lung adenocarcinoma, #8 DNA methylation, #9 histone methylation, and #10 cell death. A detailed inspection of keyword bursts and the temporal evolution of research topics offers funding agencies insights into the dynamic landscape of "epigenetic modifications and PTMs in ferroptosis". This allows for strategic allocation of funds to the most promising and impactful research areas. Funding agencies can utilize this information to identify and support high-impact projects, ensuring that their resources are invested in areas with the greatest potential for scientific breakthroughs and societal benefits. It also contributes to promoting collaborations between leading researchers and institutions.

### Key Genes and Pathways

**Figure 9:**
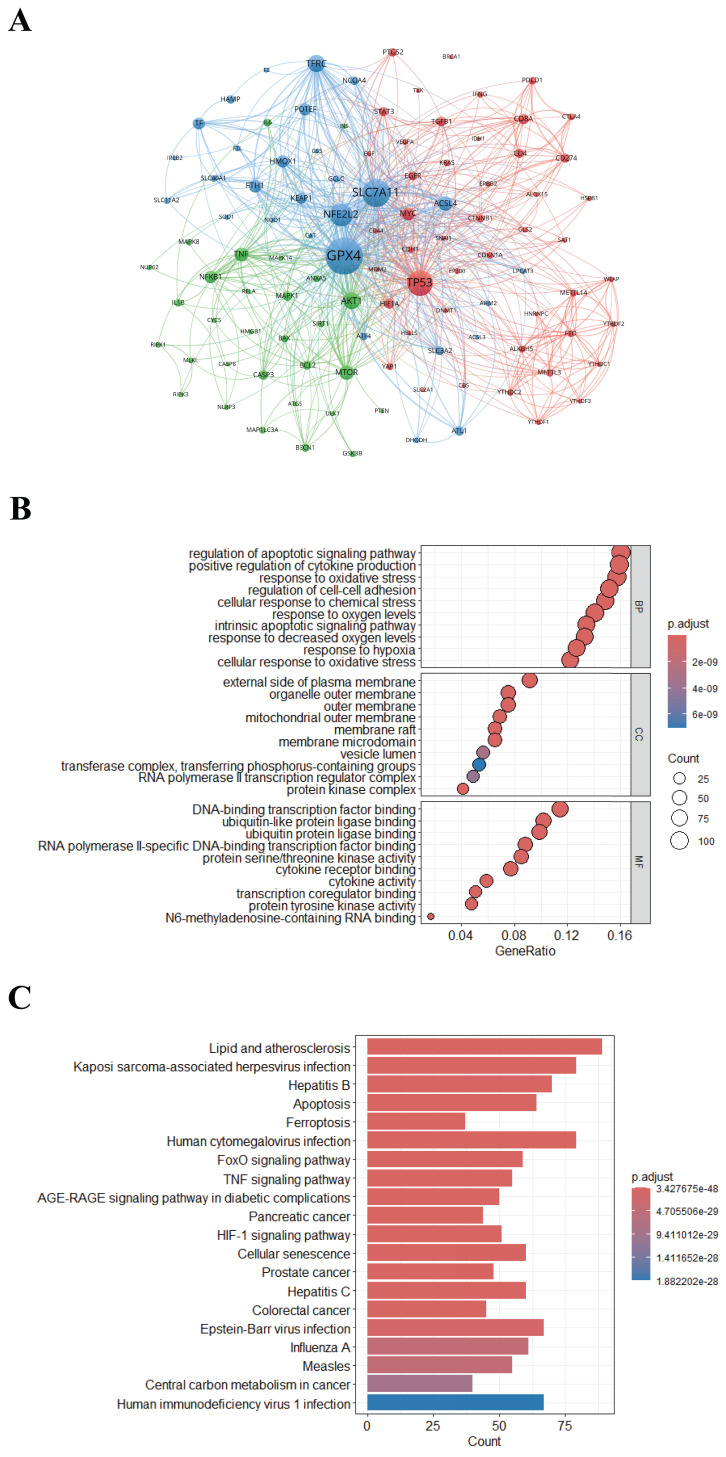
(A) VOSviewer visualization of critical gene clustering. Spheres and labels constitute nodes, with sphere size positively associated with gene occurrence frequency. Line thickness between spheres relates to the strength of gene relationships. Different colors represent clusters in different biological or medical fields. (B) Bubble plots of Gene Ontology (GO) enrichment analysis. Each bubble represents a GO term, with its size reflecting the number of genes involved and its color indicating the enrichment level. The X-axis presents the GeneRatio, which is the proportion of genes associated with the GO term in relation to the total number of genes in the background gene set (human genome). A higher GeneRatio on the X-axis implies more genes associated with the GO term. The Y-axis represents GO terms, with each node corresponding to a biological process, molecular function, or cellular component. (C) KEGG pathway enrichment analysis. The top 10 signaling pathways are presented in a bar chart. The X-axis represents the number of significantly enriched genes within each pathway, and the Y-axis lists the various pathways. Longer bars indicate a greater number of genes in that particular entry. Red indicates significantly enriched pathways, and blue indicates pathways with lower significance.

The frequency of gene occurrences and their associations provide insights into emerging research areas and changing patterns. Using VOSviewer software, we performed a co-occurrence clustering analysis of genes associated with "epigenetic modifications and PTMs in ferroptosis", thereby facilitating the identification of key genes crucial to ferroptosis research. From a comprehensive dataset consisting of 1,049 articles sourced from the Citexs big data platform, we extracted 4,224 genes, each with a citation frequency of at least 25 occurrences, and presented them in a visual map (Figure 9A). The mapping classifies genes based on their functional similarities or their co-occurrence patterns in the research literature. Each node is presented as a labeled circle, with the size of the circle corresponding to the frequency of the gene. The thickness of the connecting lines indicates the strength of associations between genes. Nodes are color-coded to distinguish distinct clusters, with each color signifying genes grouped according to specific biological or medical classifications. These clusters thereby enable researchers to identify significant genes and their roles regarding "epigenetic modifications and PTMs in ferroptosis".

Genes related to "epigenetic modifications and PTMs in ferroptosis" are crucial for understanding the changing research patterns and shifts in research focus. As shown in Figure 9A, the top three genes with the highest citation frequencies are glutathione peroxidase 4 (GPx4), solute carrier family 7 member 11 (SLC7A11), and tumor protein p53 (TP53). As a key regulator of ferroptosis, GPx4 is involved in lipid and amino acid metabolism, influencing cell aging, oncogenesis, and cell death. Recent research has highlighted the potential of treatments related to ferroptosis-related diseases^47^. Studies suggest that GPx4 can undergo various PTMs such as ubiquitination, succination, phosphorylation, and glycosylation, which influence its protein levels and activity. Altering these processes could offer therapeutic approaches for ferroptosis-related diseases^22^, ^48^. SLC7A11's expression and activity are strictly regulated by various mechanisms, including transcriptional regulation by epigenetic factors and post-transcriptional modifications (such as acetylation, methylation, ubiquitination, and phosphorylation), to control its mRNA levels, protein stability, subcellular localization, and transporter activity^49^, ^50^. TP53 is a well-known tumor suppressor protein with diverse functions, including cell cycle arrest, senescence, cell death, DNA damage repair, and mitophagy^51^. Recent evidence indicates that ferroptosis plays a crucial role in TP53-mediated tumor suppression^52^. TP53 regulates ferroptosis by directly downregulating SLC7A11 expression, thereby promoting ferroptotic cell death in response to various ferroptosis-inducing agents^53^.

We also carried out GO and KEGG enrichment analyses on signaling pathways related to "epigenetic modifications and PTMs in ferroptosis", focusing on genes cited in the articles at least twice (Figure 9B and 9C). By utilizing this data, researchers have the capacity to identify specific functions and pathways influenced by genes, which can be beneficial for experimental design and the development of hypotheses. Enhanced collaboration between researchers and industry professionals can be facilitated by identifying key genes and pathways, leveraging academic insights to accelerate the development of novel treatments or interventions. In the GO enrichment analysis bubble chart, each bubble represents a GO term, whose size reflects the number of associated genes and whose color indicates the degree of enrichment. The GeneRatio, displayed on the X-axis, represents the proportion of genes linked to a particular GO term relative to the total gene count, and higher GeneRatios indicate greater significance. The Y-axis classifies GO terms into biological processes (BP), molecular functions (MF), and cellular components (CC). As shown in Figure 9B, the GO functional enrichment results include BP, MF, and CC. In the BP category, genes are enriched in processes such as the regulation of apoptotic signaling pathways, the positive regulation of cytokine production, and the response to oxidative stress. For CC, genes are enriched in functions related to the outer side of the plasma membrane and the outer membrane of organelles. For MF, genes are enriched in functions such as binding to DNA transcription factors, binding to ubiquitin-like proteins, and binding to ubiquitin protein ligases. Analysis of enriched KEGG pathways identified the top 20 signaling pathways, which are visually represented in the histogram. The X-axis shows the number of significantly enriched genes in each pathway, while the Y-axis lists the various signaling pathways. The height of each bar indicates the number of genes within the pathway and their significance level after enrichment. Figure 9C emphasizes the significant association of this research area with signaling pathways such as 'Lipid Metabolism and Atherosclerosis' and 'Kaposi Sarcoma-Associated Herpesvirus Infection', providing valuable insights for professionals in the field regarding potential drug development opportunities in related therapeutic areas.

### Related Diseases

**Figure 10:**
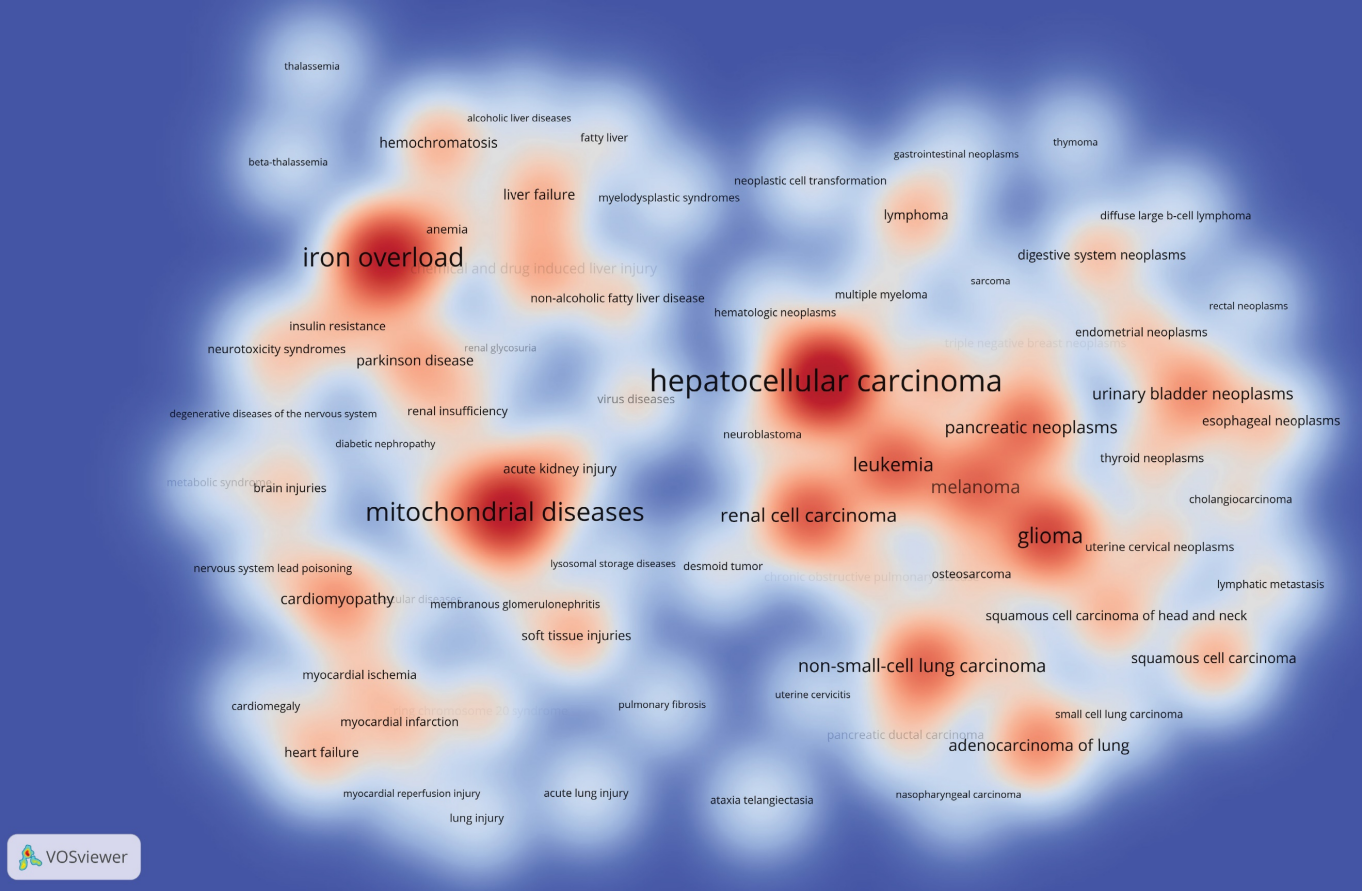
Density visualization map of related diseases. Color depth is in proportion to the frequency of disease occurrence.

Understanding diseases related to "epigenetic modifications and PTMs in ferroptosis" can guide funding allocation and prioritize research on high-impact health conditions. Policymakers can utilize this information to develop healthcare policies to enhance diagnosis, treatment, and prevention strategies for diseases linked to ferroptosis. Supporting research and development in this area can result in innovative therapies that address unmet medical needs and improve public health outcomes. The Citexs Data Platform identified 1,305 related diseases across 1,349 documents, with a threshold demanding a minimum of 15 documents mentioning each disease for inclusion. Diseases meeting these criteria were presented in a heatmap generated using VOSviewer, depicting the frequency and associations of diseases related to "epigenetic modifications and PTMs in ferroptosis" (Figure 10). The top five most frequently mentioned diseases are hepatocellular carcinoma, mitochondrial diseases, iron overload, glioma, and renal cell carcinoma.

### Challenges & Perspectives

"Epigenetic modifications and PTMs in ferroptosis" are an emerging field still in its early stages. To fully realize its potential, many questions related to epigenetic studies of ferroptosis remain unanswered^10^. Firstly, contemporary research has focused on several crucial ferroptosis-related molecules, including GPx4 and SLC7A11. It remains to be determined whether epigenetic mechanisms affect multiple ferroptosis genes and how these mechanisms interact with various signaling pathways to shape cell responses to ferroptosis stimuli^23^. Hence, a comprehensive investigation into the epigenetic regulatory network governing ferroptosis is still necessary. Additionally, in the context of ferroptosis, epigenetic regulatory pathways seldom act independently; instead, multiple pathways cooperate to regulate this process. Various epigenetic modifications can regulate the same molecule^54^, and a single epigenetic regulator may target several ferroptosis-related molecules^55^, ^56^. Consequently, these epigenetic regulatory pathways form a complex network that governs ferroptosis. However, the interactions among different epigenetic modifications and the mechanisms by which they jointly affect ferroptosis are not well understood. Additionally, studying the cell specificity of ferroptosis mediated by epigenetic modulators can help reduce clinical drug side effects. Moreover, the mechanisms by which epigenetic modifications and PTMs regulate the expression of ferroptosis-related genes in various diseases remain poorly understood. Systematic and comprehensive research is essential to determine whether novel epigenetic alterations and PTMs exhibit specificity across different diseases and cell types.

### Strengths & Limitations

In contrast to previous research that relied heavily on narrative evaluations^10^, ^16^, ^19^-^27^, this study utilized scientometric tools to obtain a comprehensive understanding of research focal areas and trends. This study is remarkable as the first in the past 12 years to undertake a scientometric analysis, mapping and describing the knowledge landscape of "epigenetic modifications and PTMs in ferroptosis". Despite inevitable limitations, it acts as a comprehensive and impartial guide for future progress.

When evaluating the study's findings, several limitations must be taken into account. Scientific mapping is a systematic and quantitative approach for assessing the structure of a knowledge base, but it does have certain drawbacks. Firstly, it should not be regarded as a substitute for review methods that assess the reliability of findings reported in academic journals, as it mainly focuses on analyzing the "meta-data" of publications. Secondly, due to the restrictions of CiteSpace, only publications from the WoSCC were incorporated, which brings about an inherent selection bias^57^, ^58^. Thirdly, the dependence on citation counts as a metric for assessing a paper's impact is exposed to various confounding factors, potentially influencing the accuracy of this evaluation^59^. Fourthly, the sheer quantity of publications may have impeded the study's completeness, restricting the ability to conduct an in-depth analysis of each paper and its subfields. Additionally, as emphasized in previous scientometric studies, the dependence of scientometric methodologies on natural language processing makes them prone to certain biases^60^, ^61^. Fifthly, this analysis took into consideration only English-language publications indexed in the WoSCC. The authors recognize that research on "epigenetic modifications and PTMs in ferroptosis" may also exist in other languages (e.g., Spanish, Chinese, French, Russian). Future research should incorporate multilingual databases to obtain more inclusive insights. Lastly, our research was restricted to academic articles available up to May 21, 2024, thereby confining our results within that specific period. However, it is important to note that this work establishes the foundation for subsequent studies, examining previous research on "epigenetic modifications and PTMs in ferroptosis".

## Conclusion

Ever since the advent of ferroptosis, there has been a significant emphasis on studying epigenetic control and PTMs. Utilizing scientometric methodologies, this study analyzes biomedical trends in "epigenetic modifications and PTMs in ferroptosis" over the past 12 years. The examination focuses on global cooperation, publishing trends, and research interest areas, providing the scientific community with insights into the development of new concepts and prospective pathways in "epigenetic modifications and PTMs in ferroptosis". For researchers, leveraging existing knowledge, seizing opportunities, conducting cross-disciplinary research, and keeping abreast of trends is of paramount importance for advancing this field.

## Supplementary Material

Supplementary information.

## Abbreviations

BP: biological processes
CC: cellular components
GO: Gene Ontology
GPx4: glutathione peroxidase 4
INSERM: Institut National de la Santé et de la Recherche Médicale
KEGG: Kyoto Encyclopedia of Genes and Genomes
LPO: lipid peroxidation
MeSH: Medical Subject Headings
MF: molecular functions
PCD: programmed cell death
PTMs: post-translational modifications
RQs: research questions
SLC7A11: solute carrier family 7 member 11
TP53: tumor protein p53
WoSCC: Web of Science Core Collection
