# Supplementary material for: Deciphering Epigenetic and Post-Translational Modifications in Ferroptosis: A Scientometric and Visualization Study
Source: Int J Med Sci. 2025 Jan 1;22(3):508–27. doi: 10.7150/ijms.104222 (PMC11783085; doi:10.7150/ijms.104222)
Supplement: Supplementary file 1 — Supplementary information. [file ijmsv22p0508s1.pdf]

Supplementary Material

1. Search strategy

Search	Query
#1	TS=("ferroptosis" OR "iron death" OR "iron overload")
#2	TS=("Epigenesis, Genetic" OR "Genetic Epigenesis" OR “Epigenetic Processes” OR "Epigenetics Processes" OR "Processes, Epigenetics" OR "Epigenetic Process" OR "Process, Epigenetic" OR "Processes, Epigenetic" OR "DNA Methylation" OR “DNA Methylations” OR “Methylation, DNA” OR “Methylations, DNA” OR “chromatin structure regulation” OR "chromatin remodeling" OR "Histone Modifications" OR “Modifications, Histone” OR "Non-Coding RNA regulation" OR "Noncoding RNA regulation" OR "ncRNA regulation" OR "Non-Coding RNA regulations" OR "Noncoding RNA regulations" OR "ncRNA regulations" OR "RNA Methylation" OR “Methylation, RNA” OR "Methylations, RNA" OR "RNA Methylations" OR “RNA Modification” OR "RNA Modifications" OR "Modification, RNA" OR "Modifications, RNA" OR "Protein Processing, Post-Translational" OR "Processing, Post-Translational Protein" OR “Post-Translational Protein Processing” OR "Post Translational Protein Processing" OR "Protein Processing, Post Translational" OR "Posttranslational Protein Processing" OR "Processing, Posttranslational Protein" OR "Protein

	Processing, Posttranslational" OR "Posttranslational Modifications" OR “Post-Translational Modifications” OR “Post Translational Modifications” OR “Protein Modification, Post-Translational” OR “Modification, Post-Translational Protein” OR "Modifications, Post-Translational Protein" OR "Post-Translational Protein Modifications" OR “Protein Modification, Post Translational” OR "Protein Modifications, Post-Translational" OR "Amino Acid Modification, Post-Translational" OR "Amino Acid Modification, Post Translational" OR "Post-Translational Amino Acid Modification" OR "Post Translational Amino Acid Modification" OR "Posttranslational Amino Acid Modification" OR " Amino Acid Modification, Posttranslational" OR “Post-Translational Protein Modification” OR "Post Translational Protein Modification" OR “Post-Translational Modification” OR "Modification, Post-Translational" OR "Modifications, Post-Translational" OR "Post Translational Modification" OR "Posttranslational Modification" OR "Modification, Posttranslational" OR "Modifications, Posttranslational" OR “Phosphorylation” OR “Phosphorylations” OR “Acetylation” OR “Acetylations” OR “ubiquitylation” OR “Ubiquitylation” OR “methylation” OR “Methylations” OR “glycosylation” OR “Protein Glycosylation” OR “Glycosylation, Protein” OR “sumoylation” OR “Sumoylations” OR “SUMO-Conjugation” OR “SUMO Conjugation” OR “SUMO-Conjugations” OR “myristoylation” OR “Lipoylation” OR “Palmitoylation” OR “Prenylation” OR “Isoprenylation” OR “Farnesylation” OR “Geranylgeranylation” OR “sulfation”)

#3	#1 AND #2
